# Hippocampal volume in early psychosis: a 2-year longitudinal study

**DOI:** 10.1038/s41398-020-00985-1

**Published:** 2020-09-01

**Authors:** Maureen McHugo, Kristan Armstrong, Maxwell J. Roeske, Neil D. Woodward, Jennifer U. Blackford, Stephan Heckers

**Affiliations:** 1 Department of Psychiatry and Behavioral Sciences, Vanderbilt University Medical Center, Nashville, TN USA; 2 Research and Development, Tennessee Valley Healthcare System, United States Department of Veteran Affairs, Nashville, TN USA

**Keywords:** Neuroscience, Schizophrenia

## Abstract

Cross-sectional studies suggest that hippocampal volume declines across stages of psychosis. In contrast, longitudinal studies indicate that hippocampal volume is stable in the critical period following illness onset. How can these seemingly disparate sets of findings be resolved? In the present study, we examine two previously unexplored reasons for this discrepancy. First, only specific subregions of the hippocampus may change during the early stage of psychosis. Second, there is diagnostic heterogeneity in the early stage of psychosis and cross-sectional analysis does not permit examination of illness trajectory. Some early stage individuals will have persistent illness leading to a diagnosis of schizophrenia, whereas in others, psychosis will remit. Hippocampal volume may be reduced only in individuals who will ultimately be diagnosed with schizophrenia. We acquired longitudinal structural MRI data from 63 early psychosis and 63 healthy control participants, with up to 4 time points per participant collected over 2 years. Subfield volumes were measured in the anterior and posterior hippocampus using automated segmentation specialized for longitudinal analysis. We observed a volume deficit in early psychosis participants compared to healthy controls that was most pronounced in the anterior hippocampus, but this deficit did not change over 2 years. Importantly, we found that anterior cornu ammonis volume is smaller at baseline in individuals who were diagnosed with schizophrenia at follow-up, but normal in those who maintained a diagnosis of schizophreniform disorder over 2 years. Smaller hippocampal volume is not diagnostic of psychosis, but is instead prognostic of clinical outcome.

## Introduction

Smaller hippocampal volume is a consistent finding in schizophrenia^1^, with the largest effect size of any brain region (Cohen’s *d* = −0.46^2^). Hippocampal volume is significantly smaller in chronic schizophrenia and early psychosis^3^, with more subtle deficits observed in individuals at clinical high risk^4,5^ and in those with genetic risk for schizophrenia^6,7^. Cross-sectional cohort studies have identified a gradient of overall volume differences in psychosis, with the largest difference (−8%) in chronic schizophrenia, moderate deficits (−4%) in the first 2 years of schizophrenia, the smallest difference (−1%) in schizophreniform disorder, and normal volume in high-risk indivduals^8–10^. This pattern suggests that hippocampal volume loss is progressive in psychosis. However, a meta-analysis of early longitudinal studies indicated a stable hippocampal volume deficit in schizophrenia^11^. Recent reports have confirmed that total hippocampal volume does not change in the first 2 years of psychosis^12–14^. Similarly, there is little evidence of progressive overall hippocampal volume loss in clinical high-risk individuals^15–17^. Taken together, there is convincing evidence that hippocampal volume loss is most pronounced in the chronic stage of schizophrenia, but no clear evidence for progressive volume loss in the first 2–5 years of psychosis.

In this study, we explore two critical gaps in the existing literature to explain this discrepancy. The first possibility is that only a part, but not all, of the hippocampus changes over time. Most previous longitudinal studies have reported total hippocampal volumes, yet the hippocampus has functionally distinct subfields and regions. The subfields (cornu ammonis, dentate gyrus, subiculum) have unique roles in learning and memory and differential vulnerability to disease^18^. The regions (anterior and posterior) have divergent patterns of connectivity^19^ and functional roles^20,21^. There is emerging evidence that the anterior but not posterior hippocampus is smaller in early psychosis^22–24^ and in clinical high-risk individuals who convert to psychosis, with the CA subfields most affected^25–28^. In contrast, anterior and posterior regions are similarly affected in chronic schizophrenia^22,24,29^. Recent longitudinal studies in psychosis have found reductions in CA1 volume over time in individuals at high risk for psychosis^26^ and in those with chronic schizophrenia^24^. However, these studies did not examine longitudinal changes in hippocampal subfields in individuals during the critical 2–5 years following illness onset^30^, nor did they examine whether changes were observed in anterior vs. posterior regions of the hippocampus. Regionally specific volume changes in early psychosis may have gone undetected in previous longitudinal studies that examined only total hippocampal volume.

A second possibility is that illness trajectory is a critical factor and hippocampal volume is reduced only in individuals who will ultimately be diagnosed with schizophrenia. A large meta-analysis of cross-sectional data found a similarly sized hippocampal volume deficit in both early and chronic schizophrenia^3^. Meta-analysis of longitudinal data suggests that illness duration may significantly impact the magnitude of observed volume deficits, particularly in the early stage of psychosis^11^. These studies collectively point to the possibility that changes in hippocampal volume occur with early illness progression.

Studies of hippocampal volume change in the early stage of schizophrenia need to take into consideration the substantial diagnostic heterogeneity in the early stages of psychosis^31^. Velakoulis and colleagues^8^ found evidence for smaller hippocampal volume in patients with early stage schizophrenia (at least 6 months, but <2 years of illness) but not in those with a diagnosis of schizophreniform disorder (<6 months of illness). This landmark neuroimaging study was an early indication that diagnostic groups, defined by partial versus full criteria set for schizophrenia (including criterion C, i.e., 6-month duration), differ in hippocampal volume. Moreover, the longitudinal studies that have examined subregional changes in hippocampal volume in early psychosis included only patients who had a diagnosis of schizophrenia^24,32^. While the majority of people with an initial diagnosis of schizophreniform disorder will have persistent illness leading to a subsequent diagnosis of schizophrenia, one-third will remit and retain the diagnosis of schizophreniform disorder^33^. Critically, no studies to date have examined how hippocampal subregion volume changes within individuals as they progress from schizophreniform disorder to schizophrenia.

Previous longitudinal investigations of hippocampal volume in the early stage of psychosis have been limited by small sample sizes, short follow-up periods, problematic measurement of hippocampal subfields^34^, and clinical heterogeneity in the period following illness onset^33^. To address the limitations of prior studies, we assessed volumes of hippocampal regions and subfields in a large sample of individuals who were in the first 2 years of a non-affective psychotic disorder. We previously established smaller volume of the anterior CA subfield in this cohort^22^. Here, we tested the primary hypothesis that anterior hippocampal volume decreases over the next 2 years of illness, with more prominent changes in the CA subfields compared to the dentate gyrus or subiculum. In a secondary analysis, we examined the impact of illness trajectory on hippocampal volume change. Two-thirds of our baseline patient sample had a diagnosis of schizophreniform disorder. We obtained 2-year follow-up scans on 89% of early psychosis participants and 83% of healthy controls with baseline data, a retention rate substantially higher than other longitudinal imaging studies in psychosis. This enabled us to test the secondary hypothesis that hippocampal volume changes would be greatest in individuals who progress to schizophrenia rather than those who maintain a diagnosis of schizophreniform disorder.

## Methods

### Participants

Participants (*N* = 137) were 72 individuals in the early stage of a psychotic disorder (EP) and 65 healthy control individuals (HC) recruited between May 2013 and September 2017 for a prospective 2-year longitudinal study on hippocampal structure and function in the early stages of psychosis (Table 1). To specifically target early pathology^35^, the majority of early psychosis participants were recruited during the initial months of illness (i.e., the average duration of psychosis was <7 months). Early psychosis participants were recruited from the inpatient and outpatient clinics of the Vanderbilt University Medical Center Psychiatric Hospital and healthy controls were recruited from the surrounding community through advertisements. Inclusion and exclusion criteria are detailed in the Supplementary Methods. Groups were recruited to be matched for mean age, gender, race, and parental education.

**Table 1:** Participant baseline demographics and clinical characteristics.

	Healthy control *N* = 63		Early psychosis *N* = 63		Healthy control > early psychosis		
	Mean	SD	Mean	SD	Statistic (*t*)	df	*p*
Age (yrs)	21.65	2.86	21.29	3.94	0.59	113.02	0.55
Parental education (yrs)	14.94	2.16	15.29	2.82	−0.78	116.12	0.44
WTAR^a^	112.07	10.53	102.77	16.31	3.72	103.03	<0.001
SCIP total Z	0.25	0.58	−0.82	0.86	8.21	108.48	<0.001
PANSS							
Positive			16.98	7.20			
Negative			17.24	8.17			
General			33.60	9.52			
Duration of psychosis (mos)			6.87	5.83			
Duration of untreated psychosis (mos)			2.04	3.80			
CPZ equivalents			311.79	149.38			

	*N*	%	*N*	%	Statistic (*Χ*^2^)	df	*p*
Gender (male)	46	73	48	76	0.17	1	0.68
Race (white)	49	78	48	76	0.04	1	0.83
Number of scans							
Baseline only	5	8	4	6			
1 Follow-up	8	13	8	13			
2 Follow-ups	7	11	18	29			
3 Follow-ups	43	68	33	52			
Diagnosis							
Schizophreniform DO			42	67			
Schizophrenia			15	24			
Schizoaffective DO			2	3			
Bipolar DO w/ psychotic features			4	6			
Current APD treatment			53	84			

*yrs* years, *mos* months, *WTAR* Wechsler Test of Adult Reading, *SCIP* Screen for Cognitive Impairment in Psychiatry, *PANSS* Positive and Negative Symptom Scale, *CPZ* chlorpromazine, *APD* antipsychotic drug, *DO* disorder, *w/* with.

^a^WTAR was unavailable for 4 healthy control and 2 early psychosis participants.

Details regarding subject attrition are included in Supplementary Figure S1. Baseline MRI scans that passed quality control were available on 63 early psychosis and 63 control participants. Fifty-six early psychosis (89%) and 52 healthy control subjects (83%) completed the study. Volumetric data from participants in this cohort have been included in a previous cross-sectional study^22^, but the longitudinal data and analyses presented here are novel. All participants provided written informed consent and received monetary compensation for their time. The Vanderbilt University Institutional Review Board approved the study.

### Clinical and cognitive characterization

We collected clinical data during in-person interviews at baseline and at the end of the study (Table 1, Supplementary Table S1). Psychiatric diagnoses were assessed with the Structured Clinical Interview for DSM-IV, TR (SCID)^36^. All data gathered during the in-person interviews were augmented by extensive review of all available medical records. Taking into account all available information, diagnostic consensus meetings were held and final diagnoses were made by psychiatrist S.H. Clinical symptoms at the time of scanning were characterized using the Positive and Negative Symptom Scale (PANSS^37^). The onset of psychosis was determined through the Symptom Onset in Schizophrenia Inventory (SOS^38^), a standardized measure for rating prodromal versus psychotic symptoms. The duration of psychosis was calculated as the amount of time between the date of onset of psychosis (determined with the SOS) and study enrollment. The duration of untreated psychosis was calculated as the time between the date of onset of psychosis (determined with the SOS) and the date of first antipsychotic treatment. Chlorpromazine equivalents were calculated using the formulas from Gardner et al.^39^. No patients treated with antipsychotic drugs at the time of study received first-generation antipsychotic medications. Premorbid IQ was estimated using the Wechsler Test of Adult Reading (WTAR^40^). Cognitive function was assessed at baseline and 2-year follow-up using the Screen for Cognitive Impairment in Psychiatry (SCIP^41^). Clinical and cognitive characteristics of the sample are described in Table 1 and the supplement.

### Data acquisition and processing

The first 2–5 years of psychosis are thought to be a “critical period” in which illness progression is most prominent and interventions are likely to have their greatest impact^30,42^. Consequently, to maximize the possibility of capturing hippocampal volume changes associated with illness progression, structural imaging data were collected up to four times over 2 years, with scans separated by ~8 months (median times to follow-up in months: follow-up 1 = 8; follow-up 2 = 16; follow-up 3 = 23). We acquired a 3D T1-weighted image on 1 of 2 identical 3T Philips Intera Achieva scanners with a 32-channel head coil (Philips Healthcare, Inc., Best, The Netherlands) at the Vanderbilt University Institute of Imaging Science (voxel size = 1 mm^3^; field of view = 256 mm^2^; number of slices = 170; gap = 0 mm; TE = 3.7 ms; TR = 8.0 ms). Each image was visually inspected for motion or other artifacts prior to inclusion (no images were removed).

**Fig. 1:**
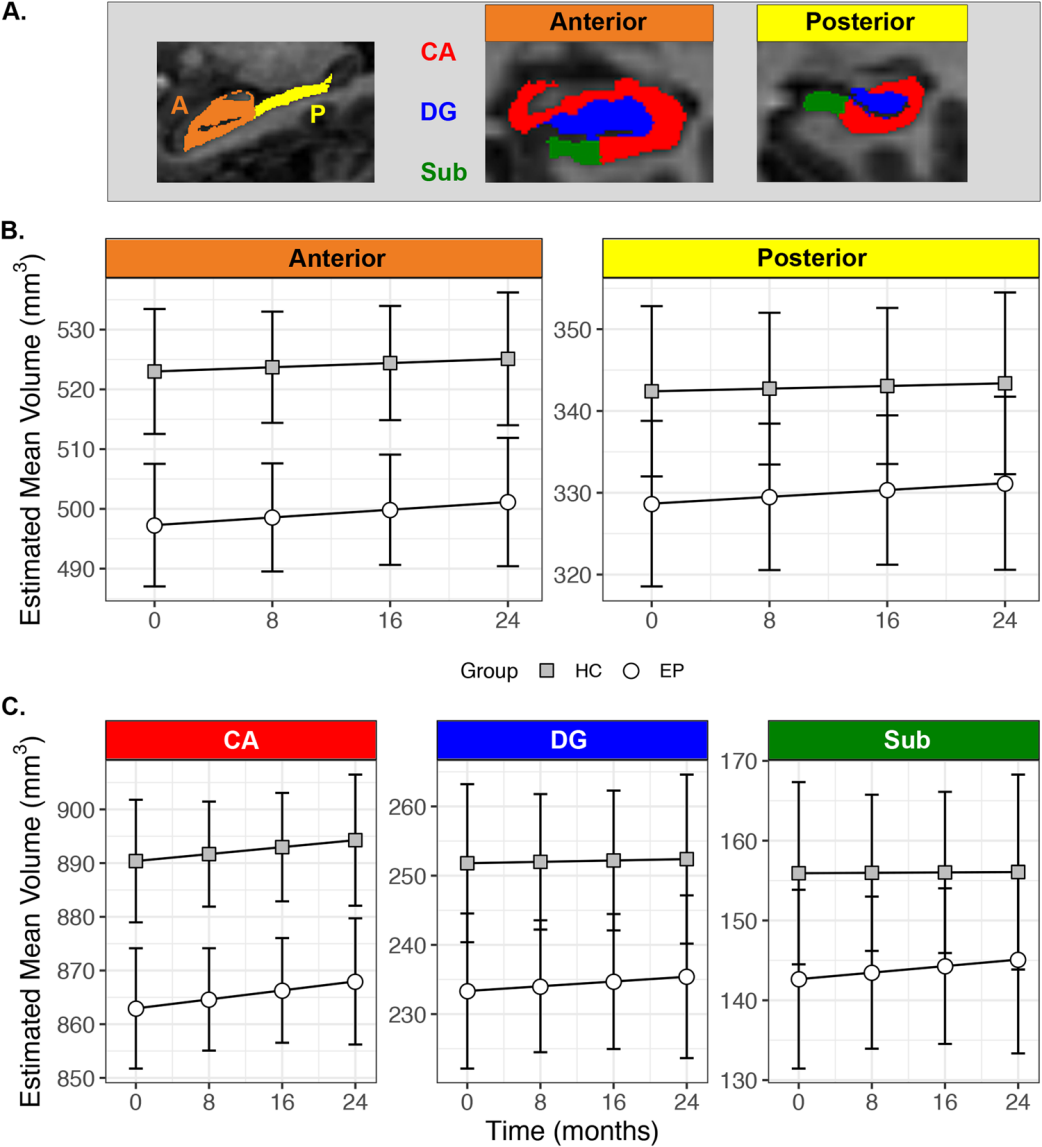
The anterior hippocampus and CA subfields are smaller in early psychosis. **a** Hippocampal regions (anterior, posterior) and subfields (CA, DG, Sub) from a single subject. **b** Early psychosis patients show a more prominent volume deficit in the anterior region than in the posterior hippocampus, but this does not change over 2 years. Circles indicate the estimated marginal mean volume of regions averaged across subfields. **c** Hippocampal subfields are differentially affected but stable in early psychosis, with the largest volume deficit in CA. Lines indicate the estimated marginal mean volume of subfields averaged across regions. Error bars indicate the 95% confidence intervals of the mean.

Structural images were processed using the Freesurfer 6 longitudinal pipeline for hippocampal subfield segmentation^43,44^. This software uses a probabilistic atlas of hippocampal subfields derived from post-mortem specimens to automatically label a T1-weighted image. The longitudinal algorithm uses a Bayesian framework to create an unbiased within-subject template of the hippocampus that minimizes within-subject error and is optimized to detect changes in volume over time. To provide generalizable results that can be better compared to findings from other hippocampal segmentation protocols^45^, we created composite subfield definitions for the cornu ammonis (CA), dentate gyrus (DG), and subiculum (Sub)^22^. The volume of each subfield was measured separately in the head (anterior) and body (posterior) regions of the hippocampus (Fig. 1a). We did not analyze data from the tail region for two reasons. Segmentation of subfields was not available for this area, as delineation can be difficult even with higher resolution imaging^44^. Moreover, our previous cross-sectional study indicated that volume differences in early psychosis are limited to more anterior regions^22^. The quality of each segmentation was assessed for inclusion of extra-hippocampal tissue or exclusion of hippocampal regions. This led to the exclusion of all data from 5 EP and 1 HC; volumes for 4 EP and 2 HC in the anterior region; and volumes for 1 EP and 1 HC in the posterior region. For analyses of regions and subfields, volumes were averaged across hemispheres to reduce the number of parameters in the statistical model.

### Statistical analysis

Statistical analyses were conducted using linear mixed models in R (R Core Team, 2019) with the packages lme4^46^, emmeans^47^, and car^48^. Models comparing EP and HC groups were fitted by adjusting for estimated intracranial volume, scanner, age at baseline, and sex, with participant as a random effect. For all models, we conducted significance tests on the fixed effects using analysis of variance (ANOVA) on the model output. Significant interactions were followed up with contrasts adjusted for multiple comparisons using Bonferroni correction. Cohen’s *d* effect sizes for follow-up contrasts were calculated using the formula contained in ref. ^49^. Prior to study initiation, we conducted a power analysis and determined that with the present sample size, we would have 80% power to detect an effect size of *d* = 0.46 in our primary longitudinal analysis. First, to test the hypothesis that there is an overall hippocampal volume deficit in early psychosis that does not change over 2 years, we constructed a model with Volume as the dependent variable and Group (EP, HC), Hemisphere (left, right), and Time in months from baseline scan as fixed effects (Model 1; Fig. S2). Second, to test whether anterior hippocampus volume decreases over 2 years of illness, with more prominent changes in the CA subfields, we fit a linear mixed model with Volume as the dependent variable and Group (EP, HC), Region (anterior, posterior), Subfield (CA, DG, Subiculum), and Time included as fixed effects (Model 2). Exploratory analyses examining the relationship between hippocampal volume and clinical and cognitive characteristics are detailed in the supplement (Supplementary analyses and results, Fig. S3).

To examine whether hippocampal volume would differ by illness course, we conducted a similar analysis to Model 2, but with groups defined by diagnostic trajectories. The illness trajectories of early psychosis participants were defined based on whether they (1) met criteria at study entry for schizophrenia (SZ stable; *N* = 16); (2) had a diagnosis of schizophreniform disorder at baseline but met criteria for schizophrenia at 2 years (SZF progression; *N* = 24); or (3) maintained a diagnosis of schizophreniform disorder over 2 years (SZF stable; *N* = 14). Illness trajectories for individual participants are depicted in Fig. 1 and summarized in Supplementary Fig. S1B. Healthy controls were included as a separate group. Only individuals who completed the study were included. Four early psychosis participants who converted from a baseline diagnosis of bipolar disorder with psychotic features to schizophrenia at follow-up were included in the SZF progression group (total SZF progression; *N* = 28). Results of the analysis did not differ when these four individuals were excluded (Trajectory × Region × Subfield interaction: F_6,2043_ = 2.17, *p* = 0.04).

## Results

### Hippocampal volume in early psychosis: effect of region and subfield

We found strong evidence of smaller left and right hippocampal volume in early psychosis participants compared to healthy control participants (Supplementary Fig. 2; main effect of Group: F_1,155_ = 13.08, *p* < 0.001; Group × Hemisphere interaction: F_1,482_ = 2.26, *p* = 0.13). This overall group difference did not change over 2 years of psychosis (Group × Time interaction: F_1,484_ = 0.26, *p* = 0.61).

**Fig. 2:**
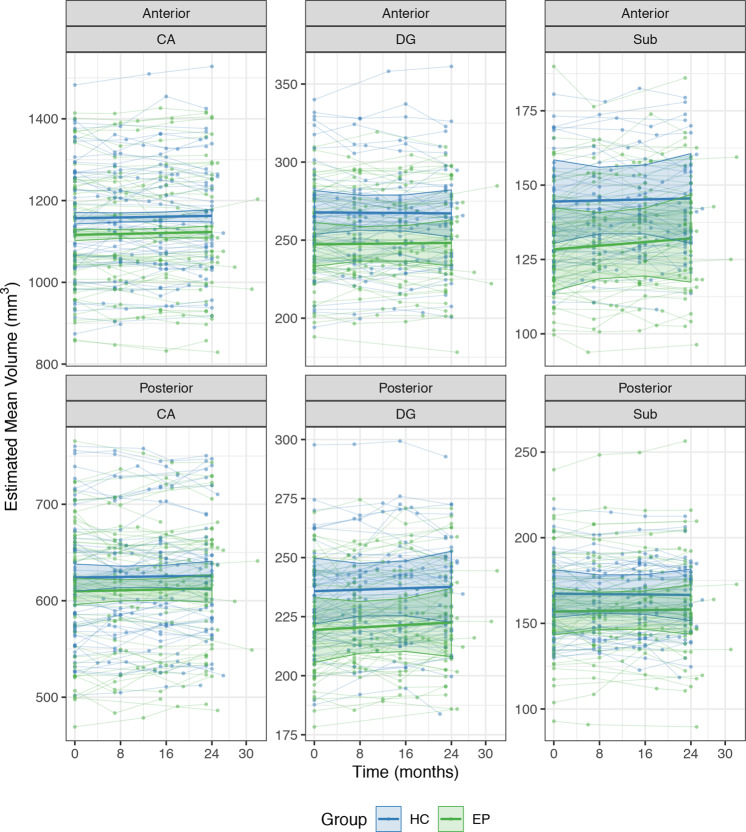
Volumes of hippocampal subfields within each region do not change over 2 years. Points and thin lines indicate raw volume data for individual participants. Heavy lines indicate the estimated marginal mean volume of subfields within each region from linear mixed model. Shaded regions indicate the 95% confidence intervals of the mean.

When we examined subregion volumes in the hippocampus, we found that volume deficits were more pronounced in the anterior than the posterior region (Fig. 1b; Group × Region interaction: F_1,1713_ = 5.36, *p* = 0.02, *d* = 0.74). This was confirmed by follow-up tests (anterior: *t*_182_ = −4.15, *p* < 0.001; posterior: *t*_179_ = −2.19; *p* = 0.06, *d* = 0.36). We observed weak evidence that hippocampal subfields are differentially affected in early psychosis (Fig. 1c; Group × Subfield interaction: F_2,1706_ = 2.50, *p* = 0.08), with greater volume differences in CA (*t*_246_ = −4.18, *p* < 0.001, *d* = 0.75) than in DG (*t*_246_ = −2.70, *p* = 0.02, *d* = 0.48) or subiculum (*t*_246_ = −1.92, *p* = 0.17, *d* = 0.30). The volumes of hippocampal regions and subfields did not change over the course of 2 years (Fig. 1b: Group × Region × Time interaction: F_1,1707_ = 0.00, *p* = 0.95; Fig. 1c: Group × Subfield × Time interaction: F_2,1706_ = 0.00, *p* = 1.00; Fig. 2: Group × Region × Subfield × Time interaction: F_2,1706_ = 0.00, *p* = 1.00).

### Hippocampal volume in early psychosis: effect of illness trajectory

**Fig. 3:**
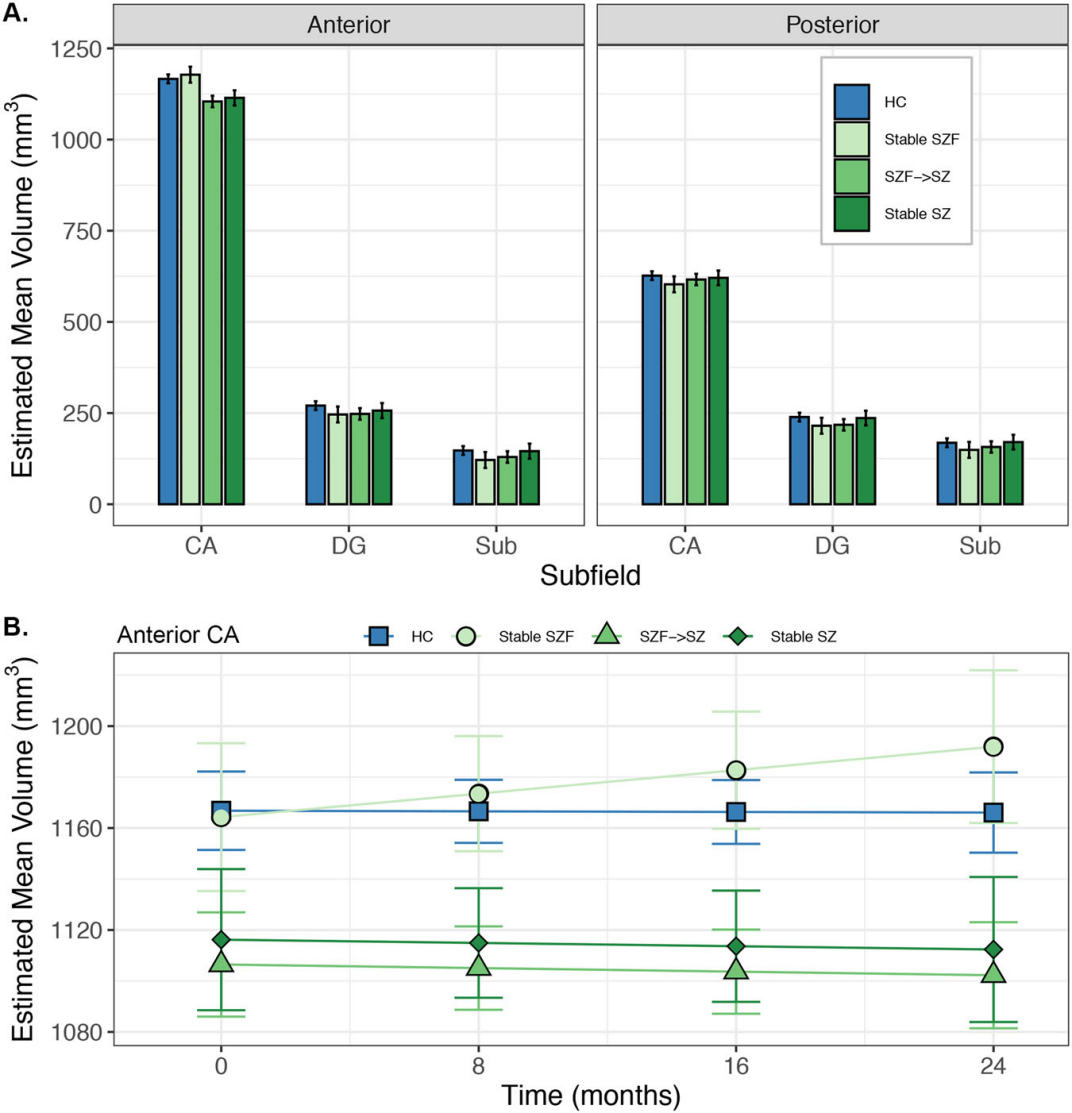
Hippocampal subregion volumes differ by 2-year diagnostic trajectory. **a** Patients with a diagnosis of schizophrenia at 2-year follow-up (SZF→SZ, Stable SZ) have selective reduction of anterior CA volume compared to healthy participants (HC). In contrast, individuals who maintain a diagnosis of schizophreniform disorder over 2 years (Stable SZF) have normal hippocampal volume across all subregions. Bars indicate the estimated marginal mean volume of subfields by region averaged across time. **b** The anterior CA volume deficit in patients with a follow-up diagnosis of schizophrenia is present at baseline and does not change over 2 years. Lines represent estimated marginal means and 95% confidence intervals for each group from a linear mixed model analyzing volume over time.

We conducted a secondary analysis to investigate whether hippocampal volume differed between early psychosis participants who did or did not progress to schizophrenia from a schizophreniform disorder diagnosis. We observed regionally specific hippocampal volume deficits that varied with diagnostic progression (Fig. 3a; Trajectory × Region × Subfield interaction: F_6,2117_ = 2.24, *p* = 0.04). Follow-up tests revealed anterior CA volume deficits in participants who met criteria for schizophrenia upon study entry (SZ stable vs. HC: *t*_312_ = −4.48, *p* < 0.001, *d* = 0.83) and in participants who were diagnosed with schizophrenia after 2 years (SZF progression vs. HC: *t*_309_ = −6.57, *p* < 0.001, *d* = 1.24). Critically, we found normal anterior CA volume in participants who maintained a diagnosis of schizophreniform disorder over 2 years (SZF stable vs. HC: *t*_316_ = 0.92, *p* = 1.0, *d* = 0). These group differences were stable over time (Fig. 3b; Trajectory × Region × Subfield × Time interaction: F_6,2117_ = 0.02, *p* = 1.0).

## Discussion

Using a longitudinal case-control design, we show that anterior hippocampal volume is smaller in early psychosis and does not change over the next 2 years of illness. We confirm that these differences are most prominent in the CA subfields compared to the dentate gyrus or subiculum. Importantly, we find that anterior CA volume is already smaller at baseline in individuals who progress to schizophrenia, but not in individuals who maintain a diagnosis of schizophreniform disorder. Our findings provide a critical bridge between prior longitudinal studies of hippocampal subregions in individuals at clinical high risk for psychosis^26^ and those in later stages of schizophrenia^24,32^.

The present study is the first to examine longitudinal changes in both anterior and posterior regions as well as hippocampal subfields in early psychosis. Our study replicates and extends findings from previous longitudinal studies that have described smaller hippocampal volume with no subsequent change over 1–2 years following the first episode of psychosis^12–14^. Our findings are consistent with the two previous studies that have examined longitudinal changes in hippocampal subregion volume in the early stage of psychosis. One early study found a stable pattern of smaller anterior hippocampus/amygdala volume^32^, but did not have sufficient resolution to disambiguate the hippocampus and amygdala. A recent study by Ho and colleagues^24^ found smaller CA1 volume in early psychosis, but did not consider anterior vs. posterior differences and included only a small number of patients in the first 5 years of illness with a longer time to follow-up (~5 years). Here, we confirm the findings from our earlier cross-sectional study^22^ using multiple within-subject measurements that provide more robust estimates of hippocampal volume: deficits in early psychosis are more pronounced in the anterior than the posterior region, and in the CA subfields than in the dentate gyrus or subiculum. Future studies using high-resolution structural imaging of the hippocampus are needed to determine whether the CA1 or CA2/3 subfields are differentially affected^50^.

Intriguingly, growing evidence suggests that this picture may look different after 5 years of illness. Previous work from our group indicates that in chronic schizophrenia, volume deficits exist throughout the hippocampal long axis^22^. Changes in the posterior hippocampus may be indicative of refractory illness^51^. The study by Ho and colleagues found that baseline volume deficits in the CA1 subfield of early psychosis individuals have spread to other subfields at 5-year follow-up^24^. These findings are bolstered by other longitudinal studies suggesting that overall hippocampal volume decline is greatest in the first 5 years of illness^11,52,53^. We did not find evidence for hippocampal pathology after 2 years of illness that reached the levels observed in chronic schizophrenia. Importantly, this suggests that the window for interventions to prevent brain changes in psychosis is still open during this period. Longer-term longitudinal studies that track brain changes in tandem with clinical and cognitive features following the onset of psychosis are needed to fully characterize the relationship between hippocampal volume loss and illness progression. Indeed, recent reports suggest that clinically distinct subgroups of schizophrenia may show differential changes in hippocampal structure^12,54^.

Our results contrast with findings from a seminal cross-sectional study of hippocampal volume across stages of psychosis^8^. That study found markedly smaller hippocampal volume in individuals with first-episode schizophrenia (<2 years of illness), but not in those with schizophreniform disorder (<6 months of illness), suggesting that hippocampal volume declines with illness progression following onset. Conversely, we observed stable, smaller anterior CA volume at baseline in individuals with schizophreniform disorder who developed schizophrenia over the next 2 years. Individuals with schizophreniform disorder who did not transition to a diagnosis of schizophrenia had normal hippocampal volume. Indeed, careful examination of anterior CA volume over time shows a tendency for individuals with a stable schizophreniform disorder diagnosis to have slightly increasing volume over 2 years (Fig. 3b). Our data indicate that hippocampal structure is already impacted in many, but not all, individuals at the first episode of psychosis, consistent with findings from individuals at high risk for psychosis^25–28^. Collectively, these data indicate that smaller volume in the anterior CA region is indicative of subsequent clinical worsening rather than current diagnostic status and are consistent with a neurodevelopmental origin of schizophrenia^55^.

The disagreement between the present findings and the study by Velakoulis and colleagues likely stems from the limitations of a cross-sectional design. The trajectories suggested by cross-sectional studies are valid only when all patients with a diagnosis are similar^56^. Approximately one-third of individuals with a diagnosis of schizophreniform disorder will not convert to schizophrenia^33^. In the schizophreniform disorder cohort examined by Velakoulis et al., heterogeneity within the patients may have simultaneously masked the true extent of volume deficits present in individuals who would later be diagnosed with schizophrenia, while overestimating the deficits present in those who did not transition to schizophrenia. Our findings suggest that the volume difference between first-episode schizophrenia and schizophreniform disorder observed in the cross-sectional study was partly due to lead time bias due to illness stage in the schizophrenia cohort^57^. This interpretation is supported by findings from a recent cross-sectional study in which patients who were in their first hospitalization for psychosis were stratified based on their diagnosis 1 year after scanning^58^. Individuals who were later diagnosed with schizophrenia showed smaller hippocampal volume and shape abnormalities in a region consistent with the anterior CA1 subfield compared to those who did not have schizophrenia.

Our study provides compelling evidence that neural structure is different already, in the early stages of an emerging psychotic disorder, between patients who will progress to schizophrenia and patients who will not progress to schizophrenia. At baseline, patients in the “stable SZF” group were matched with the “SZF→SZ” group on many variables that are associated with hippocampal volume differences, including symptom severity (PANSS positive, negative, and general scores), use of antipsychotic medication, CPZ equivalent dosage, and current cognition (Supplementary Results and Table S1). Despite being matched on all of these variables, the SZF→SZ group had lower hippocampal volume than the SZF stable group already at baseline. This finding strongly supports the argument that there are important neural differences between individuals who maintain a diagnosis of schizophreniform disorder and those who ultimately receive a diagnosis of schizophrenia. Indeed, the only discernible clinical or cognitive factor separating these 2 groups of patients at baseline was premorbid IQ: the stable SZF group had a premorbid IQ comparable to healthy controls while the SZF→SZ group had a lower premorbid IQ. Our data point to the importance of cognitive reserve as a protective factor in staving off progression to schizophrenia^59^. A critical future step is to understand convergent changes in other structures known to be affected in schizophrenia, particularly the prefrontal cortex^60^.

The main strengths of our study include dense sampling of hippocampal volume over 2 years of psychosis and a very low attrition rate in both psychosis and control groups. There were several limitations to the present study. First, we enrolled individuals with psychosis predominantly in the first 6 months of illness, but the majority of these people were medicated (Table 1). Secondary analyses indicated that chlorpromazine (CPZ) equivalent dosage was not related to anterior CA volume (Supplementary Analyses, Table S2). However, treatment with antipsychotic drugs may lead to loss of brain volume^61–64^, and thus we cannot completely rule out the confounding effects of medication in our data. Next, our primary case/control analyses included relatively large, well-matched groups, but our secondary analyses involved a smaller group of individuals with stable schizophreniform disorder. While the sample size for this group was comparatively small, we had an average of 3 scans on these individuals, supporting the stability of our estimates of hippocampal volume. In addition, our psychosis sample has a higher proportion of males than is present in the schizophrenia population^65^. This gender distribution may have led to worse outcomes in the current sample. Larger scale studies are needed to confirm the present results. Finally, we have focused on the hippocampus because of its hypothesized role in driving the pathophysiology of psychosis^66,67^.

Our findings provide unique insight into hippocampal structure during the critical period of time following psychosis onset. Compromised volume of the anterior CA region may be an important predictor of clinical progression across the psychosis spectrum.

## Supplementary information

Supplementary Information

Supplementary Tables
