# Supplementary material for: Hippocampal volume in early psychosis: a 2-year longitudinal study
Source: Transl Psychiatry. 2020 Sep 1;10:306. doi: 10.1038/s41398-020-00985-1 (PMC7463254; doi:10.1038/s41398-020-00985-1)

**Supplementary Information**

**Supplementary methods**

Early psychosis participants were recruited from the outpatient clinics and inpatient units of the Vanderbilt University Medical Center Psychiatric Hospital. Inclusion criteria were: 1) age 13 – 40; 2) estimated premorbid IQ greater than or equal to 75; 3) < 2 years of psychotic illness; and 4) meeting criteria A for schizophrenia [1, 2]. Additionally, only participants meeting criteria for either schizophreniform disorder, schizoaffective disorder, or schizophrenia at 2-year follow-up were included in this analysis. Early psychosis participants were excluded if they reported active substance abuse or dependence in the past month or if a psychotic disorder due to a medical condition was diagnosed. Healthy control participants were recruited from the community via advertisement and included if they did not meet criteria for any Axis I disorder at enrollment and at the end of the study, and did not have a first-degree relative with a known psychotic disorder. Exclusionary criteria for all participants included the presence of significant head injury, major medical illnesses, or pregnancy.

In our secondary analysis, we considered only participants who completed the 2-year follow-up visit. We operationalized illness trajectory based on the participant diagnosis at study entry compared to the diagnosis after 2 years. The illness trajectories of early psychosis participants were defined based on whether they (1) met criteria at study entry for schizophrenia (SZ stable; N=16); (2) had a diagnosis of schizophreniform disorder at baseline but met criteria for schizophrenia at 2 years (SZF progression; N=24); or (3) maintained a diagnosis of schizophreniform disorder over 2 years (SZF stable; N=14).

**Supplementary analyses and results**

*Clinical and cognitive characteristics – overall sample*

Early psychosis and healthy control participants were matched on all demographic factors (age, parental education, gender, and race; Table 1, main text). At baseline, early psychosis participants overall had lower estimated premorbid IQ (WTAR: t_103_=3.72, p<0.001) and lower current cognitive functioning as measured by the SCIP (t_108_=8.21, p<0.001).

*Clinical and cognitive characteristics by illness trajectory*

Demographic and clinical characteristics of the sample who completed 2-year follow-up are presented in Supplementary Table S1. The groups defined by illness trajectory were matched on demographic factors including age, parental education, gender, and race. Consistent with our characterization of illness trajectories, at baseline, the early psychosis groups defined in this manner had similar levels of psychosis symptom severity (main effect of Group, PANSS positive: F_2,55_=0.60, p=0.55; PANSS negative: F_2,55_=0.16, p=0.85; PANSS general: F_2,55_=1.32, p=0.28). In contrast, the illness trajectory groups differed after 2 years in positive (main effect of Group: F_2,54_=10.16, p<0.001) and general (main effect of Group: F_2,54_=6.22, p=0.01), but not negative symptoms (main effect of Group: F_2,54_=2.97, p=0.06). Follow-up tests confirmed that the stable SZF group had lower levels of positive and general symptoms than either the SZF progression (PANSS positive: t=-3.04; p=0.01; PANSS general: t=-2.97, p=0.01) or stable SZ groups (PANSS positive: t=-4.48, p<0.001; PANSS general: t=-3.29, p=0.01). Illness trajectory groups did not differ on CPZ equivalents of antipsychotic dosage at baseline (F_2,45_=2.71, p=0.08) or at 2-year follow-up (F_2,33_=2.23, p=0.12). Similarly, the groups did not differ in the number of medicated individuals at baseline (Χ_2_^2^=1.34, p=0.51) or at 2-year follow-up (Χ_2_^2^=5.42, p=0.07).

The illness trajectory groups differed on estimated premorbid IQ (main effect of Group: F_3,102_=5.57, p=0.001). The SZF stable group had similar IQ to healthy controls (t=0.19, p=0.84), whereas the SZF progression and stable SZ groups had lower IQ than controls (SZF progression: t=3.63, p<0.001; stable SZ: t=2.50, p=0.01). Illness trajectory groups differed on current cognition both at baseline (main effect of Group: F_3,106_=19.42, p<0.001) and at 2-year follow-up (main effect of Group: F_3,104_=14.75, p<0.001). At baseline, all early psychosis patients had lower current cognitive function than healthy controls (stable SZF: t=3.60, p=0.003; SZF progression: t=5.74, p<0.001; stable SZ: t=6.28, p<0.001), but were not different from each other (all p’s > 0.33). At 2-year follow-up, the SZF stable group had similar current cognitive function to healthy controls (t=2.16, p=0.20), whereas the SZF progression and stable SZ groups had lower current cognitive function than controls (SZF progression: t=5.58, p<0.001; stable SZ: t=5.08, p<0.001).

*Association of anterior hippocampal volume with clinical and cognitive factors in early psychosis*

We carried out additional exploratory analyses to examine whether the anterior CA volume deficit observed in the present study was associated with clinical characteristics of our patient sample. Because we did not observe any changes over time in our longitudinal analyses, we first examined the association between average baseline anterior CA volume and clinical characteristics that have been previously reported to be related to hippocampal volume in psychosis [3–5] We conducted linear regression analyses to test for an association between positive, negative, and general PANSS scores, chlorpromazine equivalents, duration of untreated psychosis, and medication status (medicated/unmedicated). We found no evidence for an association between any of the clinical characteristics examined and anterior CA volume (Supplementary Table S2). In the present study, we identified early psychosis participants at or very soon after their first psychotic episode. It is possible that the restricted range for some variables in our sample have may obscured evidence of a relationship with hippocampal volume. For example, the majority of individuals in our study had a duration of untreated psychosis of less than 2 months and were medicated.

To identify whether there were associations between changes in clinical and cognitive characteristics and anterior CA volume over time in individuals with psychosis, we conducted additional linear mixed models to test for longitudinal relationships between medication status (yes/no) and positive, negative, and general symptoms measured by PANSS subscale scores. We did not find evidence for a relationship between medication status (F_1,97_=0.03, p=0.86), PANSS negative (F_1,131_=0.50, p=0.48), or PANSS general scores (F_1,131_=0.52, p=0.47) and anterior CA volume over time. We observed a significant interaction between PANSS positive scores and time in association with anterior CA volume (interaction: F_1,131_=8.91, p=0.003). To better understand this interaction, we conducted a simple effects analysis examining the effect of different quartiles of PANSS positive scores (scores = 9, 13, and 17) estimated at times 0, 8, 16, and 24 months (Supplementary Figure S3). Anterior CA volumes did not differ between levels of positive symptoms at times 0 (t=-2.0, p=0.11), 8 (t=-0.19, p=0.98), or 16 months (t=1.46, p=0.31). At 24 months, there was a nonsignificant trend for lower positive symptoms to be associated with higher anterior CA volume (t=2.17, p=0.08). This finding is similar to the apparent trend for individuals with a stable schizophreniform disorder diagnosis to have slightly increasing anterior CA volume over 2 years (main text, Figure 3B). The anterior hippocampus, and the CA1 subfield in particular, increase in volume during development, reaching their peak in late adolescence / early adulthood [6, 7]. The interaction we observed may reflect normative hippocampal development in individuals with minimal positive symptoms and slightly decreased hippocampal volume in those with higher levels of positive symptoms [8]. Overall cognitive function was not correlated with hippocampal volume across trajectory groups (Group X Volume interactions for total, anterior, and anterior CA volume p’s > 0.1).

**References**

1. First M, Spitzer R, Miriam G, Williams J. Structured Clinical Interview for DSM-IV-TR Axis I Disorders, Research Version, Patient Edition with Psychotic Screen (SCID-I/P W/PSY SCREEN). 2002.

2. Diagnostic and statistical manual of mental disorders: DSM-5^TM^, 5th ed. Arlington, VA, US: American Psychiatric Publishing, Inc.; 2013.

3. Ho NF et al. Progression from selective to general involvement of hippocampal subfields in schizophrenia. Mol Psychiatry. **22**, 142–152 (2017).

4. Van Erp TGM et al. Subcortical brain volume abnormalities in 2028 individuals with schizophrenia and 2540 healthy controls via the ENIGMA consortium. Mol Psychiatry. **21**, 547–553 (2016).

5. Goff DC et al. Association of Hippocampal Atrophy With Duration of Untreated Psychosis and Molecular Biomarkers During Initial Antipsychotic Treatment of First-Episode Psychosis. JAMA Psychiatry. **75**, 370–378 (2018).

6. Schlichting ML, Guarino KF, Schapiro AC, Turk-Browne NB, Preston AR. Hippocampal Structure Predicts Statistical Learning and Associative Inference Abilities during Development. J Cogn Neurosci. **29**, 37–51 (2017).

7. Tamnes CK, Bos MGN, van de Kamp FC, Peters S, Crone EA. Longitudinal development of hippocampal subregions from childhood to adulthood. Dev Cogn Neurosci. **30**, 212–222 (2018).

8. Kalmady SV et al. Clinical correlates of hippocampus volume and shape in antipsychotic-naïve schizophrenia. Psychiatry Res - Neuroimaging. **263**, 93–102 (2017).

**Supplementary Figures**

**Supplementary Figure S1**. (A). Participant attrition for longitudinal sample. (B). Diagnostic trajectories for patients. Abbreviations: F/U = follow-up; SUD = substance use disorder; BP = bipolar disorder with psychotic features; SZF = schizophreniform disorder; SZA = schizoaffective disorder; SZ = schizophrenia.

**Supplementary Figure S2.** Hippocampal volume is reduced in early psychosis in the left and right hemispheres compared to healthy control participants and does not change over 2 years. Error bars indicate the 95% confidence intervals of the mean.


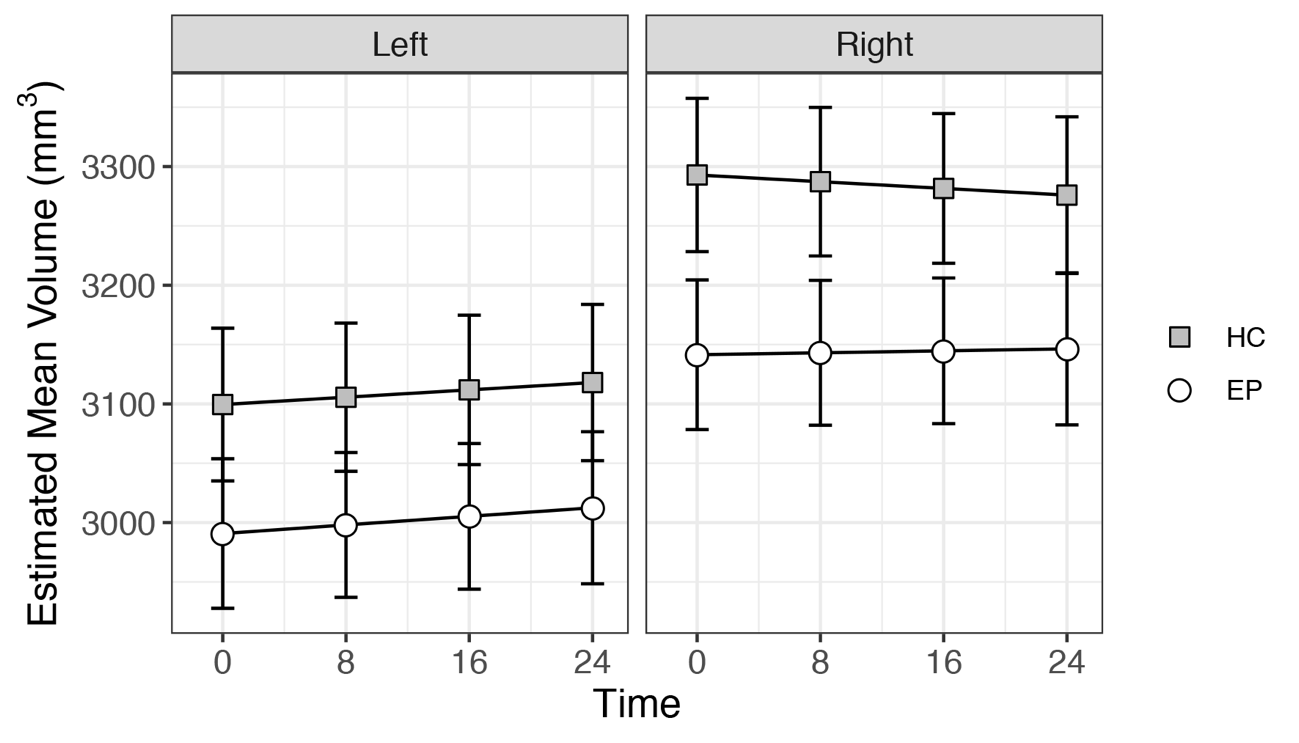


**Supplementary Figure S3.** Anterior CA volume over 2 years in individuals with differing levels of average positive symptoms measured by the PANSS positive subscale (PANSS_P).


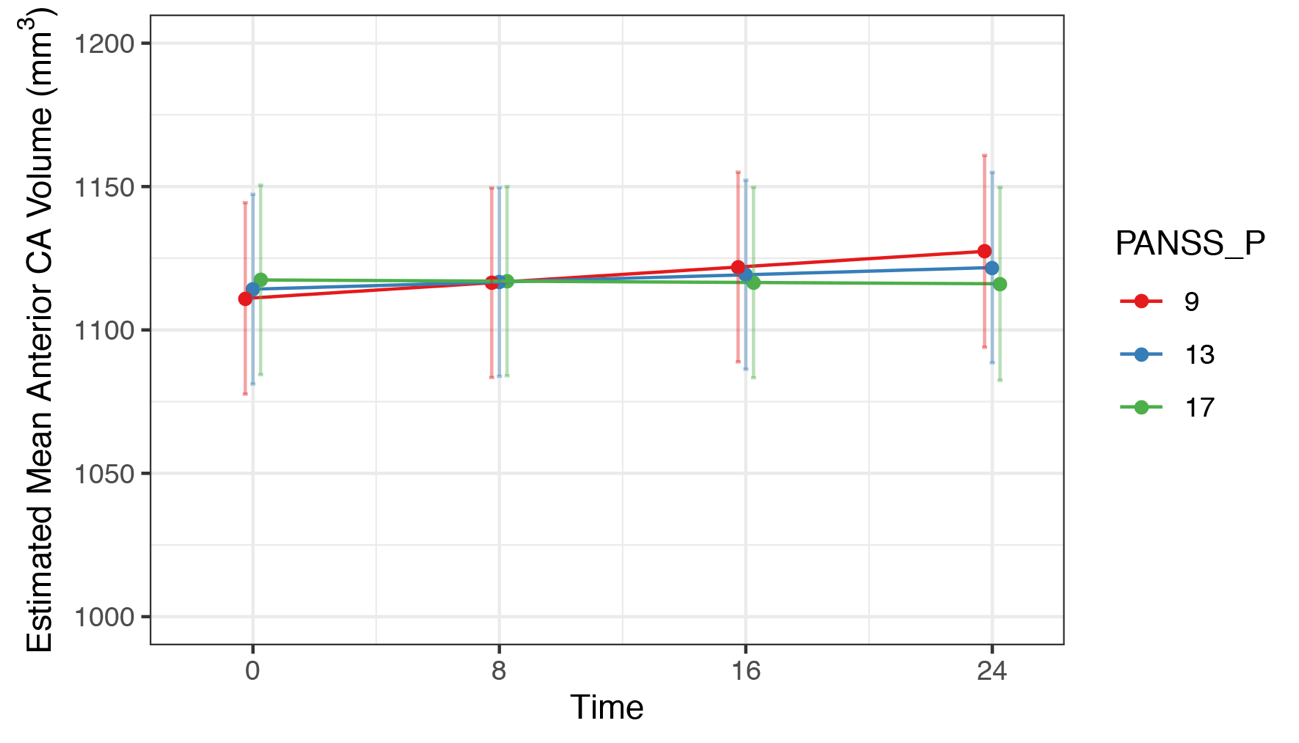

Supplement: Supplementary file 1 — Supplementary Information [file 41398_2020_985_MOESM1_ESM.docx]
