# Supplementary material for: Hippocampal volume in early psychosis: a 2-year longitudinal study
Source: Transl Psychiatry. 2020 Sep 1;10:306. doi: 10.1038/s41398-020-00985-1 (PMC7463254; doi:10.1038/s41398-020-00985-1)
Supplement: Supplementary file 2 — Supplementary Tables [file 41398_2020_985_MOESM2_ESM.docx]

**Supplementary Table S1.** Demographic and clinical information by 2-year diagnosis.

|  | **Healthy Control** | | **Stable Schizophreniform** | | **Schizophreniform 🡪 Schizophrenia** | | **Stable Schizophrenia** | |  |  |  |  |
| --- | --- | --- | --- | --- | --- | --- | --- | --- | --- | --- | --- | --- |
|  | N=52 | | N=14 | | N=28 | | N=16 | |  |  |  |  |
|  | **Mean** | **SD** | **Mean** | **SD** | **Mean** | **SD** | **Mean** | **SD** | **Statistic (F)** | **p** |  |  |
| **Age** (yrs) | 21.48 | 2.95 | 20.71 | 3.73 | 20.64 | 4.00 | 23.12 | 3.84 | 1.95 | 0.13 |  |  |
| **Parental Education** (yrs) | 14.88 | 2.14 | 16.21 | 3.26 | 15.09 | 2.50 | 15.06 | 3.09 | 1.02 | 0.39 |  |  |
| **WTAR^1^** | 111.44 | 10.84 | 110.64 | 11.22 | 99.56 | 18.22 | 101.33 | 15.11 | 5.57 | 0.001 |  |  |
| **Time since onset** (mos) | |  | 6.78 | 8.19 | 4.52 | 4.28 | 11.35 | 4.10 |  |  |  |  |
| **Duration of untreated psychosis** (mos) | | | 3.50 | 6.94 | 0.85 | 0.76 | 2.14 | 2.51 |  |  |  |  |
|  | **N** | **%** | **N** | **%** | **N** | **%** | **N** | **%** | **Statistic (Χ^2^)** | **p** |  |  |
| **Male** | 40 | 77 | 11 | 79 | 23 | 82 | 11 | 69 | 1.06 | 0.79 |  |  |
| **Race** (White) | 40 | 77 | 13 | 93 | 21 | 75 | 11 | 69 | 2.68 | 0.44 |  |  |
| **Number of scans^2^** |  |  |  |  |  |  |  |  |  |  |  |  |
| Baseline only | 0 | 0 | 1 | 7 | 0 | 0 | 0 | 0 |  |  |  |  |
| 1 Follow-up | 6 | 11 | 1 | 7 | 5 | 18 | 2 | 13 |  |  |  |  |
| 2 Follow-ups | 3 | 6 | 3 | 21 | 9 | 32 | 4 | 25 |  |  |  |  |
| 3 Follow-ups | 43 | 83 | 9 | 64 | 14 | 50 | 10 | 63 |  |  |  |  |
|  | **Baseline** | **Follow-up** | **Baseline** | **Follow-up** | **Baseline** | **Follow-up** | **Baseline** | **Follow-up** | **Baseline**  **Statistic** | **Baseline**  **p** | **Follow-up**  **Statistic** | **Follow-up**  **p** |
| **SCIP Total Z^3^** | 0.25 ± 62 | 0.38 ± 0.55 | -0.54 ± 0.67 | -0.12 ± 0.63 | -0.73 ± 0.94 | -0.64 ± 1.00 | -1.05 ± 0.67 | -0.73 ± 0.97 | F=19.42 | <0.001 | F=14.75 | <0.001 |
| **PANSS** |  |  |  |  |  |  |  |  |  |  |  |  |
| Positive |  |  | 16.14 ± 6.75 | 9.07 ± 2.16 | 15.93 ± 7.27 | 13.19 ± 4.77 | 18.25 ± 6.78 | 15.81 ± 4.15 | F=0.60 | 0.55 | F=10.16 | <0.001 |
| Negative |  |  | 15.86 ± 5.63 | 8.93 ± 3.15 | 16.68 ± 9.01 | 13.15 ± 6.54 | 17.56 ± 8.47 | 14.06 ± 7.46 | F=0.16 | 0.85 | F=2.97 | 0.06 |
| General |  |  | 32.21 ± 8.34 | 21.42 ± 2.90 | 31.64 ± 10.81 | 27.67 ± 6.75 | 36.31 ± 7.55 | 29.13 ± 7.78 | F=1.32 | 0.28 | F=6.22 | 0.01 |
| **CPZ equivalents** |  |  | 225 ± 136 | 135 ± 65 | 318 ± 171 | 322 ± 229 | 363 ± 106 | 355 ± 182 | F=2.71 | 0.08 | F=2.23 | 0.12 |
| **Current APD treatment** | |  | 10 | 5 | 23 | 19 | 15 | 12 | Χ^2^=1.34 | 0.51 | Χ^2^=5.42 | 0.07 |

^1^ WTAR was unavailable for 2 Healthy Control, 1 Schizophreniform🡪Schizophrenia, and 1 Stable Schizophrenia.

^2^ Follow-up scan data was unavailable for 1 Stable Schizophreniform due to subsequent metal exclusion for MRI. Two-year follow-up data was excluded for 1 Schizophreniform 🡪Schizophrenia because of current substance use disorder.

^3^ SCIP was unavailable at follow-up for 1 Healthy Control and 1 Schizophreniform🡪Schizophrenia.

Abbreviations: yrs=years; mos = months; WTAR=Wechsler Test of Adult Reading; SCIP = Screen for Cognitive Impairment in Psychiatry; PANSS = Positive And Negative Symptom Scale; CPZ = Chlorpromazine; APD = Antipsychotic Drug.

**Table S2.** Association of clinical characteristics with baseline mean anterior CA volume in early psychosis participants.

|  | **Statistic** | **p** |
| --- | --- | --- |
| **PANSS** |  |  |
| Positive | t = -0.87 | 0.39 |
| Negative | t = 0.22 | 0.83 |
| General | t = -0.13 | 0.90 |
| **Duration of untreated psychosis** (months) | t = -0.93 | 0.36 |
| **Chlorpromazine equivalents** | t = -0.71 | 0.49 |
| **Medicated vs. Unmedicated** | t = 0.50 | 0.62 |
